# Impact of Comorbidity of Bronchial Asthma and Type 2 Diabetes Mellitus on the Expression and Functional Activity of TLR2 and TLR4 Receptors

**DOI:** 10.3390/life13020550

**Published:** 2023-02-16

**Authors:** Yaroslav V. Radzyukevich, Ninel I. Kosyakova, Isabella R. Prokhorenko

**Affiliations:** 1 Hospital of Pushchino Scientific Center, Russian Academy of Sciences, Pushchino 142290, Russia; 2 Department of Molecular Biomedicine, Institute of Basic Biological Problems, Federal Research Center “Pushchino Scientific Center for Biological Research of the Russian Academy of Sciences”, Pushchino 142290, Russia

**Keywords:** bronchial asthma, type 2 diabetes mellitus, comorbidity, toll-like receptors, lipopolysaccharides, lipoteichoic acid

## Abstract

Epidemiological data indicate the active progression of various forms of diabetes mellitus in patients with bronchial asthma (BA), but little is known about the mechanisms of comorbidity formation. TLR2 and TLR4 are involved in the progression of asthma and type 2 diabetes mellitus (T2DM). These receptors are involved in the inflammatory response to Gram(+) and Gram(−) bacteria, respectively, so changes in their expression may affect the predisposition of patients to bacteremia. The aim of this study was to analyze the expression and functional activity of toll-like receptor 2 and 4 (TLR2 and TLR4) on peripheral blood cells of patients with BA, T2DM, and BA + T2DM. The expression of TLR2 and TLR4 was analyzed by flow cytometry. Whole blood samples were incubated with lipopolysaccharides from *E. coli* (LPS) and lipoteichoic acid from *S. pyogenes* (LTA). The concentration of cytokines and soluble blood proteins was determined by ELISA. Patients with comorbid diseases showed a statistically significant increase in TLR2 expression on both monocytes and neutrophils compared with healthy donors and patients with BA. We found increased expression of TLR4 on the surface of blood monocytes from patients compared to donors. The activation of blood cells of patients and donors with LPS or LTA led to an increase in the expression of “fast” pro-inflammatory cytokines (TNF-α, IL-6). In patients with BA, the average production of TNF-α in response to endotoxin was two times higher than in other studied groups. The reactions of blood cells in patients with T2DM and BA + T2DM did not differ significantly. The expression and functional activity of TLR2 and TLR4 on the blood cells of patients with comorbid disease were similar to those only in patients with T2DM. The greatest increase in the synthesis of the pro-inflammatory cytokine TNF-α in response to LPS and LTA was observed in patients with BA, which can lead to an inadequate response to bacteremia.

## 1. Introduction

The activation of TLR2 and TLR4 receptors could underlie the pathophysiological mechanisms of many human diseases including bronchial asthma (BA), type 2 diabetes mellitus (T2DM), obesity, metabolic syndrome, and autoimmune diseases [1,2,3].

Asthma is a heterogeneous disease characterized by chronic inflammation and hyperreactivity of the respiratory tract. Type 2 diabetes mellitus (T2DM) is a multifactorial disease characterized mainly by decreased insulin sensitivity, impaired insulin secretion, and chronic inflammation [4].

Epidemiological data and conventional medical practice reveal the active progression of various types of diabetes mellitus (DM) in patients with bronchial asthma [5]. A statistical analysis demonstrated a significant correlation between carbohydrate metabolism and the risk of bronchial asthma progression [6]. In cases of asthma and type 2 diabetes mellitus (T2DM), the vitally essential role of obesity and insulin resistance was shown in the stimulation of an excessive pro-inflammatory immune response [7,8].

Nowadays, more and more attention is paid to the role of sex and gender in the development, formation, and treatment susceptibility of asthma and T2DM. On the one hand, women suffer from T2DM more often [9], and they have more frequent comorbid diseases and a more severe effect of risk factors, such as obesity [10,11]. On the other hand, the risk of obesity-related asthma is also higher in women. Meanwhile, women with asthma have a higher occurrence of dyslipidemia, diabetes mellitus type 2, and hypertension than men, although the reasons remain unclear [12]. The main causes of sex-related differences are the changes in sex hormone concentrations that could lead to the effect of age-dependent transitions, such as puberty in men and women, and the menstrual cycle, pregnancy, and menopause in women, on asthma and diabetes. Sex differences could also be related to genetic, cellular, or physiological aspects, but the studies in these directions are in progress [13,14].

The recent studies show the effect of weak systemic inflammation and oxidative stress on the association between asthma and diabetes mellitus [8]. Although the role of TLRs in infectious pathologies is well-known, more and more data give evidence on the key role of these receptors in the progression and stabilization of chronic inflammation [15].

Systemic chronic inflammation accompanying T2DM and asthma could lead to the decreased functional activity of immune cells, which increases the risks of both Gram-positive and Gram-negative bacteremia.

From the theoretical point of view, the possible etiological mechanisms of comorbidity of the two diseases and factors affecting their mutual course should be found out. On the one hand, TLR2 and TLR4 are involved in the progression of asthma and T2DM. On the other hand, they participate in the inflammatory response to Gram(+) and Gram (-) bacteria, respectively, so changes in their expression during BA or T2DM could affect the susceptibility of patients to bacteremia. Beyond all the aforementioned factors, the comorbid state of patients complicates the selection of an effective treatment regime. From the point of view of practice, the data on the peculiarities of the comorbid state and differences between T2DM/BA and BA +T2DM will allow healthcare practitioners to reveal the specific features of the clinical picture and to select the appropriate treatment schedules in case of comorbidity of these diseases.

The goal of the study was an analysis of the expression and functional activity of TLR2 and TLR4 on the cells of peripheral blood of patients with bronchial asthma (BA), type 2 diabetes mellitus (T2DM), and a combination of both diseases (BA + T2DM).

## 2. Materials and Methods

The object of the study involved obtaining the peripheral blood of 27 patients from the immunological and allergological departments of the Hospital of Pushchino Scientific Centre RAS, who had a confirmed diagnosis of “Bronchial asthma” (BA, 9 patients), “Type 2 Diabetes mellitus” (T2DM, 11 patients), or “BA + T2DM” (7 patients). The mean age of the patients was 64 ± 3 years. Cytokine content before and after the promotion of blood cells by *E. coli* endotoxin or lipoteichoic acid from *S. pyogenes* was determined. The control group included donors (n = 11) without BA or T2DM in anamnesis. The study was approved by the Local Ethical Committee of HPSC RAS, protocol number 9 from 12 July 2017. Blood samples were taken during remission phases of the diseases; the exacerbation of the diseases (T2DM and/or BA) was absent according to a clinical laboratory analysis during the last four weeks before blood acquisition [16].

### 2.1. Determination of TLR2 and TLR4 Expression

Blood was taken from the basilic vein, and heparin was used as the anti-coagulant. The following tagged antibodies were used: IgG2a Alexa Fluor^®^ 488 (BioLegend, San Diego, CA, USA), TLR2 Alexa Fluor^®^ 488 (Invitrogen, Carlsbad, CA, USA), and TLR4 Alexa Fluor^®^ 488 (eBioscience, San Diego, CA, USA). Blood samples were incubated with corresponding antibodies for 30 min in the dark. RBC Lysis Buffer (BioLegend, San Diego, CA, USA) was used for disintegration of red blood cells. The samples were analyzed by flow cytofluorimeters Epic XL-MCL (Beckman Coulter, Brea, California, USA) and NovoCyte (Agilent, Santa Clara, CA, USA). In total, 50,000 events in each region (or 1500 events in monocyte region) were registered. Depending on size and granularity, groups of neutrophils and monocytes were distinguished (Supplementary Materials).

Data analysis and processing were performed in Flowing Software and FloReada programs. Expression levels of the studied receptors were determined by a change of fluorescence intensity (MnIX, a.u.) The values were normalized to the autofluorescence of non-tagged cells. The significance of differences was calculated according to non-parametric Mann–Whitney criterion.

### 2.2. Whole Blood Promotion to Cytokine Synthesis

The S-glycoform of *E. coli* O55:B5 LPS and lipoteichoic acid of *Streptococcus pyogenes* were used in the work (Sigma-Aldrich, Merck, Saint Louis, MI, USA). RPMI1640 growth media (Sigma-Aldrich, Merck, Rahway, NJ, USA) was used for cell incubation. Heparinized blood (5% heparin concentration) was diluted with RPMI 1640 medium at 1:9 ratio and aliquoted in 1 mL portions in 48-well plates (“Cellstar”) (Greiner Bio-one, Kremsmünster, Austria). To study the effect of cell promotion by endotoxins, *E. coli* LPS (100 ng/mL) or *Streptococcus pyogenes* LTA (1000 ng/mL) was added to the wells. The samples were incubated for 6 or 24 h at 37° in 5% CO_2_ atmosphere in a CO_2_ incubator (“Biosan”, Latvia). After the incubation, the cells were sedimented by centrifugation for 10 min at 1000 rpm on a special centrifuge LMC-3000 (“Biosan”, Latvia). The obtained supernatants were collected and stored at −20 °C until the cytokine determination analysis.

### 2.3. Determination of Cytokine Content

A concentration of TNFα, IL-6, IL-1β, IL-8, and IL-10 was determined using solid-state ELISA kits (Vector-Best AO, Novosibirsk, Russia). ELISA kits were also used to determine soluble blood proteins LBP, sCD14 (Hycult Biotech, Uden, The Netherlands), and sMD-2 (MyBioSource, San Diego, CA, USA).

### 2.4. Statistical Data Analysis

The results are presented as median values with an interquartile range (IQR). The significance of differences between median values was estimated by the Mann–Whitney U-test and Wilcoxon signed-rank test. The presence of correlations was estimated by Pearson’s rank test. Differences of median values were considered as significant at *p* < 0.05. Program packages Microsoft Office Excel 2010 (AtteStat plugin), STATISTICA 10.1, and SigmaPlot 12.5 were used for the statistical analysis and graphical data representation.

## 3. Results

### 3.1. Characterization of Patient Groups

All the studied patients had approximately the same age, weight, and height, and had an elevated BMI. Patients with T2DM had specific high glucose concentration and a high level of glycated hemoglobin in blood, while in patients with BA + T2DM, these values were slightly elevated, but close to normal ones. Asthma in anamnesis predictably decreased FEV1, although the value was just above the minimal normal level. The patients were in the remission stage. The basic characteristics of the studied patient groups are listed in Table 1.

The presence of C-reactive proteins in the blood of patients with T2DM in anamnesis attracted attention, while this protein was virtually absent in patients with BA. This could point to the presence of weak chronic inflammation during T2DM, while in the remission stage of BA, inflammation was apparently absent.

### 3.2. Expression of TLR2 Receptor

The TLR2 receptor is involved in the pathogenesis of BA and T2DM. Upon binding of a ligand, TLR2 forms a heterodimer with TLR1 or TLR6, activating intracellular signal transduction pathways and cytokine expression [17].

The level of expressed TLR2 was significantly variable depending on cell type, and the maximal level was expressed on the monocyte surface, whereas neutrophils expressed TLR2 in at least 4-fold-less amounts (see Table 2).

Lymphocytes displayed only slight expressions of TLR2 and TLR4 (Table 3).

**Figure 1:**
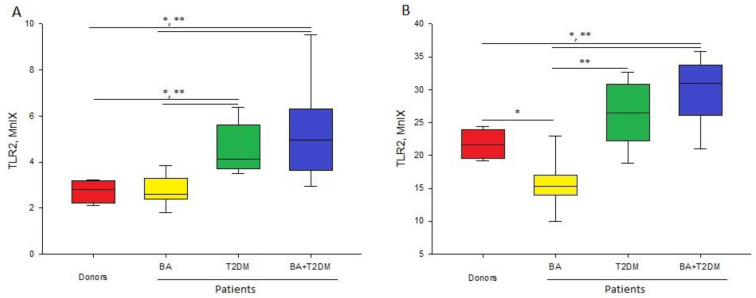
Level of fluorescence intensity of TLR2 (MnIX) on neutrophils (**A**) and monocytes (**B**) in patients and healthy donors; *—significant (*p* < 0.05) difference from healthy donors; **—significant difference from patients with BA.

Patients with comorbid diseases showed statistically reliable elevations of the expression of TLR2 on both monocytes and neutrophils compared to healthy donors and patients with BA. Patients with T2DM in anamnesis did not show changes in the expression of TLR2 on monocytes compared to healthy donors, but this value was significantly higher than in patients with BA. Such a change was related to the reliable decrease in this receptor level in patients with BA compared to other groups. It should be noted that there were no statistically significant differences between patients with T2DM in anamnesis, despite the tendency of elevated expressions of TLR2 in cases of comorbid diseases (Figure 1).

There are contradictory data in the literature on the changes of expression of TLR2 in patients with BA. A number of authors showed that the surface monocyte TLR2 expression is elevated in patients with BA, which is related to the pre-activated state of monocytes and pro-inflammatory state of the immune system [18,19]. On the other hand, there are data on the absence of significant differences in the surface expression of this receptor in healthy donors and patients with BA [20]. The difference in results could be explained by the high mobility of changes in the surface TLR2 level depending on the state of patients and the treatment received. The decrease in expression of this receptor observed in this work could be related to effective anti-inflammatory treatment received by the patients.

### 3.3. Expression of TLR4 Receptor

The TLR4 receptor forms a homodimer and activates intracellular signal transduction that induces the synthesis of pro- and anti-inflammatory cytokines upon ligand binding [21].

Our results show that TLR4 expression on monocytes was more active than on neutrophils. The level of TLR4 expression was significantly lower than that of TLR2 (Table 3).

The mean values of TLR4 expression on neutrophils differed in the studied groups, but the observed tendencies did not reach statistical significance because of high intragroup variability.

**Figure 2:**
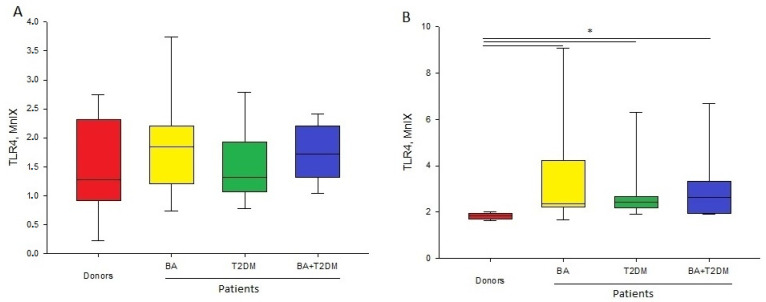
Level of fluorescence intensity of TLR4 (MnIX) on neutrophils (**A**) and monocytes (**B**) in patients and healthy donors; *—significant (*p* < 0.05) difference from healthy donors.

We found a more enhanced expression of TLR4 on the surface of the monocytes of patients from the studied groups compared to healthy donors. Patients with T2DM in anamnesis expressed more TLR4 than patients with BA only. The observed differences did not reach statistical significance. Increased TLR4 expression could point to the increase in background inflammation upon T2DM in anamnesis and increased sensitivity to pro-inflammatory stimuli (Figure 2).

Earlier in the literature, the expression of TLR receptors, including TLR2 and TLR4, in patients with T2DM in anamnesis was shown [22]. Currently obtained results confirm these data. Since these receptors are involed in the inflammatory response, one can suppose that the appearance of an additional comorbid disease leads to the elevation of patient sensitivity to Gram-positive and Gram-negative infections. Furthermore, elevated expression of these receptors could lead to an inadequate immune response to other pro-inflammatory stimuli.

### 3.4. Influence of Endotoxin and Lipoteichoic Acid on the Synthesis of Cytokines by Blood Cells

To estimate the functional activity of TLR2 and TLR4 and to test the adequacy of the immune response to Gram-positive and Gram-negative infection, we studied the change of the level of pro-inflammatory cytokine and chemokine production by the cells from patients and healthy donors in response to pro-inflammatory stimuli. For this purpose, lipoteichoic acid from S.pyogenes and lipopolysaccharide from *E. coli* were used.

Only trace amounts of pro-inflammatory cytokines (TNF-α, IL-6, IL-1β, IL-8) were detected in the control non-stimulated samples, which could demonstrate an absence of a significant inflammatory process in patients of both groups. Upon the action of LPS from *E. coli* or LTA from *S. pyogenes*, the blood cells of healthy donors and patients from both groups produced a significant amount of pro-inflammatory cytokines.

**Figure 3:**
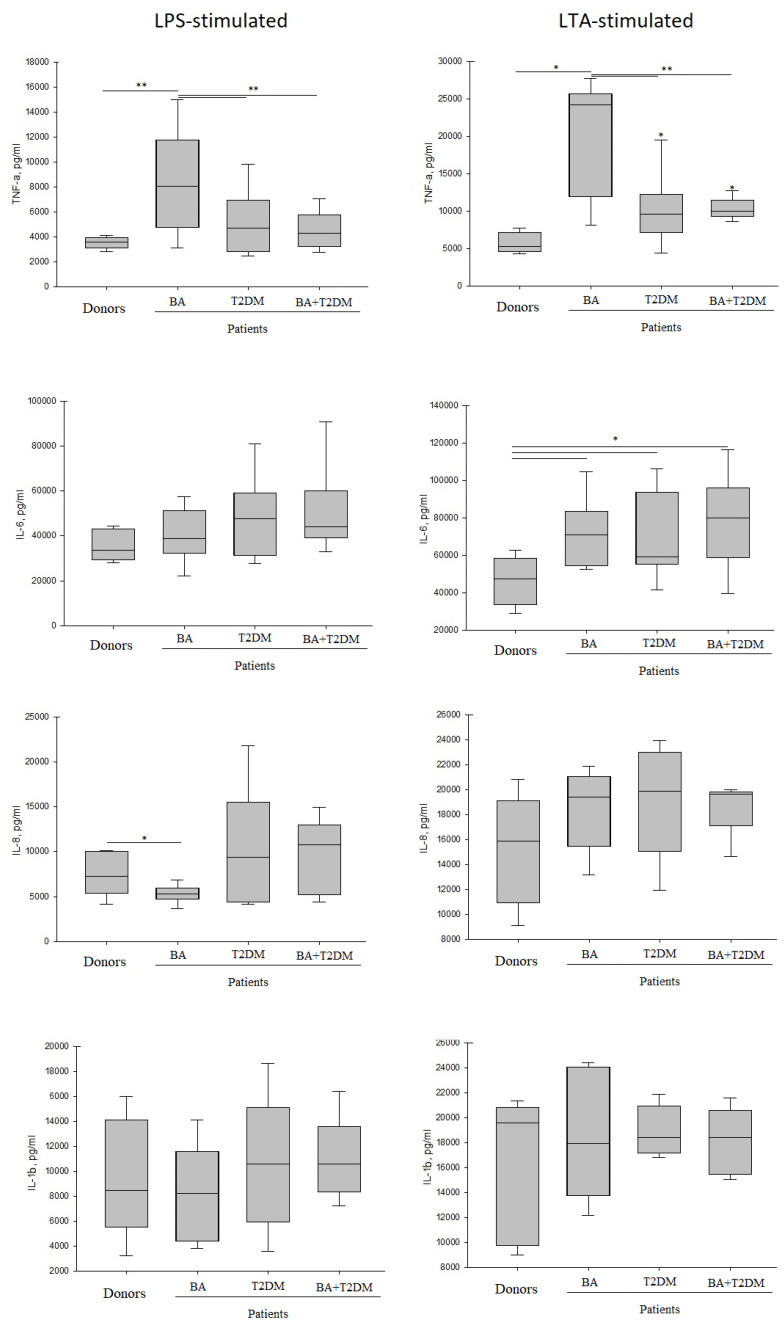
Expression of pro-inflammatory cytokines (TNF-α, IL-6, IL1β, IL-8) by whole blood cells of donors and patients with BA, T2DM, and BA + T2DM promoted by LPS and LTA. The results are presented as median values with interquartile ranges (IQR). *—significant (*p* < 0.05) difference from healthy donors; **—significant difference from patients with BA.

A significantly higher amount of cytokines was synthesized in response to LTA than in response to the endotoxin. LPS is considered as a more potent activator than LTA, and that is why the researchers use 100–1000 higher amounts of LTA than LPS [23,24,25]. The most frequently used LPS concentration is 100 ng/mL [25,26]. We used a ten-fold ratio of the concentration of pro-inflammatory agents, but observed only a two–three-fold difference in the cytokine synthesis (see Figure 3). One may suppose that the observed difference in the quantity of cytokines produced by the cells is related not only to the concentrations of LPS and LTA used, but also to the capacities of effector cells or other factors (number of free surface and intracellular receptors, possibilities of activation of intracellular signaling pathways, TLR2-TLR6 binding constant, or TLR4 dimerization parameters).

The activation of blood cells of patients with T2DM and BA + T2DM by endotoxin did not alter the expression of TNF-α compared to healthy donors. In patients with BA, the mean production of TNF-α in response to endotoxin was two times higher than in all other studied groups (see Figure 1).

**TNF-α**

After the stimulation of blood cells by *S. pyogenes* LTA, the statistically significant enhancement of TNF-α cytokine synthesis was observed in all the groups of patients compared to healthy donors. Patients with BA demonstrated high variability in TNF-α expression in response to LTA, while patients with T2DM in anamnesis displayed a significantly lower level of this receptor in response to LTA than patients with BA, and its intragroup variability was significantly lower. The blood cells of patients with T2DM expressed twice as much TNF-α as healthy donors’ cells, but these were still two-fold lower than in patients with BA. Comorbidity did not significantly alter the TNF-α level compared to T2DM-only patients. Thus, the following tendency was observed in the response of the blood cells of patients and healthy donors to LPS and LTA: the presence of T2DM in anamnesis significantly decreases the expression of the rapid pro-inflammatory cytokine TNF-α level compared to BA in response to the pro-inflammatory agent from either Gram-positive or Gram-negative bacteria. The responses of blood cells from patients with BA + T2DM were close to the responses of the cells from patients with T2DM only.

**IL-6**

Somehow, similar tendencies were not observed in the IL-6 synthesis. The blood cells of patients synthesized more IL-6 in response to LPS or LTA than the blood cells of healthy donors. Under endotoxin promotion, the observed differences did not reach statistical significance due to the high intragroup variability of the results, whereas under the action of LTA, the differences between patients and healthy donors in IL-6 synthesis were statistically significant. The responses of cells from different groups of patients did not differ significantly from each other.

**IL-8**

A significantly lower amount of chemokine IL-8 was expressed in patients with BA compared to conditionally healthy donors. Such a response might give evidence of the formation of a Th2-dependent asthma variant rather than a neutrophil-dependent one in the patients. The Th2 phenotype is generally harder to control, and it reacts to the treatment with more poor results. A non-Th2 phenotype of severe asthma, which occurs because of a neutrophil immune response, is also related to the resistance to corticosteroids, which complicates selection of a treatment scheme [27].

**IL-1β**

The synthesis of IL-1β in response to LPS and LTA did not differ in patients and healthy donors. The observed absence of differences could be related to the more complex and multistage synthesis of this “slow” cytokine, which distinguishes it from “fast” ones, which are synthesized in 4–8 h after stimulation, such as pro-inflammatory cytokines TNF-α and IL-6.

Thus, the activation of blood cells of patients and conditionally healthy donors by LPS or LTA led to the increased expression of “fast” pro-inflammatory cytokines (TNF-α, IL-6). IL-1β, which has a prolonged expression, did not display differences between the patient groups and control group. Since TNF-α is the first cytokine synthesized in response to pro-inflammatory agents, playing an important role of the mediator of rapid progression of an inflammatory response, one can suppose that patients with BA in anamnesis are the most sensitive to bacterial infection, and they could react to it with an inadequate force. The responses of blood cells from patients with T2DM and BA + T2DM did not differ significantly.

### 3.5. Role of the Expression Level of TLR2 and TLR4 Receptors in Cytokine Synthesis

The functional activity of the receptors lies in the signal transduction from the surface ligand to the cell nucleus, and, in the end, in the activation of cytokine synthesis. Based on the mechanisms of signal transduction and the activation of cytokine synthesis, the presence of correlations between receptor quantity and level of cytokine synthesis in patients and healthy donors can be supposed. The main producers of pro-inflammatory cytokines (TNF-α, IL-6, IL-1β) are monocytes [28]. We performed a correlation analysis of the levels of expression of TLR2 and TLR4 on the monocytes and synthesis of pro-inflammatory cytokines in response to LTA from *S. pyogenes* and LPS from *E. coli*, respectively.

The ratio of the quantity of the receptors on the blood cell surface and cytokines synthesized by the cells of patients varied in wide ranges inside the groups, explaining the low correlation between these parameters (Table 4).

Table 4 shows that the correlation coefficients in donors and patients were not very high. The obtained results show the lack of dependency between TLR2 expression and synthesis of the studied cytokines in response to LTA. In healthy donors, a strong direct effect of the level of TLR2 expression on IL-6 synthesis was shown, but this value did not reach statistical significance.

The expression of TLR4 on neutrophils did not significantly change, which was reflected in the expression of IL-8. We showed a strong negative correlation between these values in patients with BA, although the expression of this chemokine in response to LPS decreased significantly (see Table 4).

All of the groups of patients expressed a significantly higher quantity of TLR4 on the monocytes, but a statistically significant enhancement of TNF-α synthesis was observed only in patients with BA, which allows us to suggest the absence of a direct correlation between TLR4 expression and pro-inflammatory cytokine synthesis in response to lipopolysaccharides.

## 4. Discussion

Asthma and T2DM are very widespread chronic diseases. Currently, they are believed to share a common pathophysiological mechanism: weak chronic inflammation [29], leading to the progression of asthma in cases of T2DM or vice versa [4]. We have not shown elevation of the C-reactive protein in patients with BA, while in patients with T2DM or BA + T2DM, the CRP levels were increased. The observed rise in CRP concentration, which does not exceed the reference value [30], could point to weak chronic inflammation in patients with T2DM in anamnesis.

Despite the widespread simultaneous progression of T2DM and BA in patients, just a limited number of studies dedicated to the development of comorbidity can be found in the literature [4]. Toll-like receptors 2 and 4 play an important role in inflammation, activating synthesis of pro-inflammatory cytokines in response to LTA and LPS, respectively.

Expression characteristics of these receptors are currently well-studied only in patients with either T2DM or BA. The enhanced expression of TLR4 and decreased expression of TLR4 were shown in PBMC of the patients with severe BA [31]; however, our results do not confirm the drop of expression of TLR4 in patients with BA. The observed differences could be reasoned by different severity degrees of asthma in the studied groups.

The functional activity of the receptors was reflected in the elevated synthesis of TNF-α by mononuclear phagocytes of the patients with severe BA in response to TLR2 and TLR4-specific ligands, but other pro-inflammatory cytokines, namely IL-6, IL-8, and IL-1b, did not display such dependency. At the same time, the T2DM group had significantly increased expression of TLR2 [32] and TLR4 [33] on the surface of PBMC and monocytes [2]. The patients with T2DM did also demonstrate the significantly enhanced expression of pro-inflammatory cytokines in reaction to LPS and LTA [2]. Our results confirm the data from the literature.

Nevertheless, the role of these receptors in the progression of comorbid disease is studied very poorly. We have studied the peculiarities of the expression and functional activity of TLR2 and TLR4 in patients with BA, T2DM, and BA + T2DM.

We have found that the characteristics of the expression and functional activity of TLR2 and TLR4 receptors in patients with comorbid disease (BA + T2DM) are close to the patients with just T2DM in anamnesis. Based on the obtained results, one can suggest that the leading disease in the case of comorbidity of BA and T2DM is exactly T2DM. The parameters of the immune activity in blood cells from patients with diabetes and BA + T2DM differ significantly from the parameters of patients with asthma alone. When selecting the treatment for patients with BA + T2DM, the main attention should be paid to type 2 diabetes mellitus.

Blood cells of patients with BA react significantly stronger to pro-inflammatory stimuli of Gram(+) and Gram(-) bacteria, so bacteremia could cause an inadequately severe response in this patients.

The specificity of the obtained data was the high variability of the values inside the groups. The test of significance of the correlation coefficients showed that only patients with BA + T2DM have a statistically significant reverse correlation between the levels of TLR4 and IL-6. All the other correlations are statistically insignificant, and they may be attributed to stochastic oscillations in the samples. There are data from the literature on the presence of a correlation between TLR2 and TLR4 expression and background cytokine content [2,34], but not in response to additional promotion by bacterial agents. We have shown that the increased expression of TLR2 and TLR4 does not lead to the proportional enhancement of synthesis of pro-inflammatory cytokines in response to additional bacterial stimuli. Blood cells have sufficient quantities of the receptors to trigger the immune response.

## 5. Conclusions

The obtained data allow us to suppose that the leading disease during comorbidity of BA and T2DM is type 2 diabetes mellitus because the peculiarities of expression and functional activity of TLR2 and TLR4 on the blood cells of patients with comorbid disease were similar to the values of the patients with T2DM only. The enhanced expression of the receptors could be related to the formation of weak chronic inflammation, which is clearly shown by a higher level of CRP in patients with T2DM, but not with BA, in anamnesis. Chronic inflammation affects the monocyte expression of TLR2 more than that of TLR4. We could not find statistically reliable correlations between the expression of the studied receptors on the surface of immune cells and synthesis of pro-inflammatory cytokines in response to bacterial ligands. The highest increase in the synthesis of pro-inflammatory cytokine TNF-α in response to LPS and LTA was observed in patients with BA, which could lead to an inadequate reaction to bacteremia.

## 6. Limitations of the Study

The study has a number of limitations. The first limitation is a small number of patients in the study groups. Furthermore, due to the small sample size, we do not consider and do not discuss the role of sex in the formation and progression of the studied diseases, although there are numerous studies confirming the effect of sex and gender on the progression of T2DM or asthma [13,14]. Finally, a clear definition of the role of the studied receptors in the formation of BA, T2DM, and their comorbidity requires knockout and/or blockage of TLR2 and TLR4 via siRNA or specific antibodies, and an extension of the experimental groups as well. In spite of all the aforementioned limitations, the presence of a statistically significant difference in TLR2 and TLR4 expression and cytokine synthesis between the studied groups of patients shows an important role of TLR2 and TLR4 in the progression of T2DM and BA, and in the comorbidity of these two diseases.

## Figures and Tables

**Table 1:** Mean values of certain parameters of the patients.

Parameters	Patients with BA	Patients with T2DM	Patients with BA + T2DM	Ref. Value
Age, years	48 ± 1.7	64 ± 2	64.9 ± 3	-
Weight, kg	69.8 ± 4.8	82.4 ± 4.1	80.7 ± 2	-
Height, cm	165.7 ± 2.6	160.5 ± 2	162 ± 2.2	-
BMI	25.3 ± 1.5	31.9 ± 1.6	30.3 ± 0.5	18.5–25
Glucose, mM	5 ± 0.2	8.25 ± 0.96	6.9 ± 0.5	3.9–6.1
Glycated HB, mM	-	7.9 ± 0.5	7 ± 0.1	6.5–7.5
Cholesterol, mM	6 ± 0.4	5.1 ± 0.3	5.56 ± 0.5	3.3–5.5
Triglycerides, mM	1.6 ± 0.15	1.4 ± 0.1	2.9 ± 1.3	0.44–2.29
HDL, mM	1.7 ± 0.07	1.3 ± 0.1	1.5 ± 0.2	0.9–3
Cholesterol LDL, mM	3.8 ± 0.2	3.23 ± 0.33	3.4 ± 0.5	1.6–3.5
CRP, mg/dL	0 ± 0.01	0.36 ± 0.17	0.27 ± 0.06	0–0.5
FEV1, %	78.8 ± 4.3	102.5 ± 2.2	81.1 ± 3.9	>80
SaO2, %	97.1 ± 0.3	96.9 ± 0.2	95.9 ± 0.4	>96

BMI, body mass index; CRP, C-reactive protein; FEV1, forced expiratory volume in 1 s; SaO2, saturation.

**Table 2:** Level of fluorescence intensity of TLR2 (MnIX) on different types of cells in patients and healthy donors.

Cell Type	Conditionally Healthy Donors	Patients with BA	Patients with T2DM	Patients with BA + T2DM
Lymphocytes	1.2	1.3	1.3	1.3
	[1.2; 1.3]	[1.2; 1.4]	[1.1; 1.6]	[1.2; 1.4]
Neutrophils	2.8	2.6	4.1 *^,^**	4.9 *^,^**
	[2.3; 3.1]	[2.4; 2.9]	[3.7; 5.6]	[3.6; 6.3]
Monocytes	21.6	15.2 *	26.4 **	30.9 *^,^**
	[19.9; 23.4]	[14.2; 16.3]	[22.2; 30.1]	[27.8; 33]

*—significant difference from conditionally healthy donors; **—significant difference from patients with BA (*p* < 0.05). n = 4–11. Autofluorescence intensity is taken as 1.

**Table 3:** Level of fluorescence intensity of TLR4 on different cell types taken from patients and healthy donors.

Cell Type	Conditionally Healthy Donors	Patients with BA	Patients with T2DM	Patients with BA + T2DM
Lymphocytes	1.2	1.2	1.1	1.1
	[1.1; 1.3]	[1.2; 1.4]	[1; 1.3]	[1.1; 1.2]
Neutrophils	1.3	1.8	1.4	1.7
	[1.2; 2.3]	[1.3; 2]	[1; 2]	[1.3; 2.2]
Monocytes	1.8	2.3 *	2.4 *	2.9 *
	[1.8; 1.9]	[2.2; 3.8]	[2.9; 10.2]	[1.9; 2.9]

*—significant difference from conditionally healthy donors (*p* < 0.05). n = 4–11. Autofluorescence intensity (without fluorescence tags) was taken for 1.

**Table 4:** Correlation coefficients between TLR2 expression and pro-inflammatory cytokines in response to LTA.

Parameters	TLR2(M)—TNF-α	TLR2(M)—IL-6	TLR2(M)—IL-1β	TLR2(N)—IL-8
Donors	0.4	0.8	−0.6	0.4
Patients with BA	0.29	0.07	0.31	0.43
Patients with T2DM	−0.2	−0.1	0.19	−0.05
Patients with BA + T2DM	−0.26	−0.09	−0.66	−0.8
Parameters	TLR4(M)—TNF-α	TLR4(M)—IL-6	TLR4(M)—IL-1β	TLR4(N)—IL-8
Donors	0.1	0.8	−0.2	0.5
Patients with BA	0.29	0.38	0.38	0.86 *
Patients with T2DM	0.1	0.06	−0.19	0.36
Patients with BA + T2DM	0.26	−0.66	−0.49	0.6

TLR2(M)—expression of TLR2 on monocytes; TLR2(N)—expression of TLR2 on neutrophils; TLR4(M)—expression of TLR4 on monocytes; TLR4(N)—expression of TLR4 on neutrophils; *—significant correlation coefficient; *p* < 0.05.

## Data Availability

The data presented in this study are available on request from the corresponding author. The data are not publicly available due to ethical reasons.

## Supplementary Materials

The following supporting information can be downloaded at: https://www.mdpi.com/article/10.3390/life13020550/s1, Figure S1: (A) Gating of different cell populations. Separation of monocyte, lymphocytes and neutrophils by size to granularity ratio (direct light scattering versus side light scattering). (B,C) Mean fluorescence intensity (MnIX) of TLR2 (B) and TLR4 (C) on the monocytes of healthy donors and patients with T2DM or BA + T2DM.
