# Supplementary figures and images for: Impact of Comorbidity of Bronchial Asthma and Type 2 Diabetes Mellitus on the Expression and Functional Activity of TLR2 and TLR4 Receptors
Source: Life (Basel). 2023 Feb 16;13(2):550. doi: 10.3390/life13020550 (PMC9965069; doi:10.3390/life13020550)

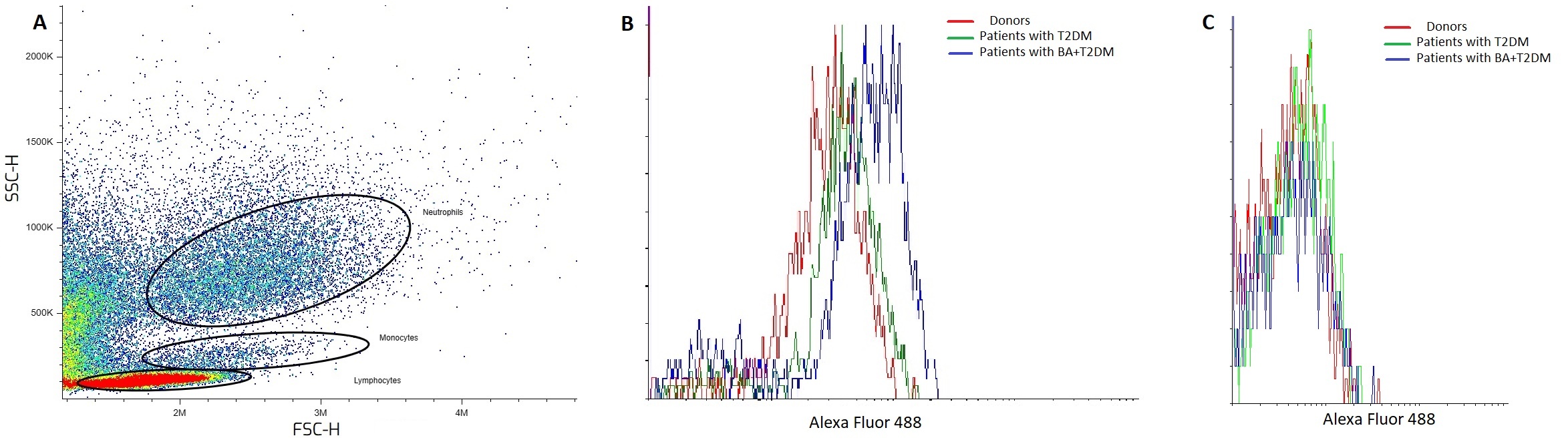

Supplement: Supplementary file 1 [file life-13-00550-s001.zip › Supplementary Figure S1.jpg]
